# Prognoses of Patients Treated With Surgical Therapy Versus Continuation of Local-Plus-Systemic Therapy Following Successful Down-Staging of Intermediate-Advanced Hepatocellular Carcinoma: A Multicenter Real-World Study

**DOI:** 10.1093/oncolo/oyad277

**Published:** 2023-10-24

**Authors:** Jianwei Liu, Xiaodong Zhu, Yangxun Pan, Jianhong Zhong, Renan Jin, Xin Zheng, Wei Zhang, Kuan Hu, Jian Ma, Xiaoyi Shi, Hongzhi Liu, Xiaobo Yang, Da Xu, Chi Ma, Jiangming Chen, Dongxu Wang, Xiaojun Wang, Zhongchao Li, Lei Zhao, Leida Zhang, Tao Li, Fubao Liu, Guang Tan, Baocai Xing, Haitao Zhao, Yongyi Zeng, Shuijun Zhang, Lei Zhang, Ledu Zhou, Tianqiang Song, Wei Yang, Xiao Liang, Bangde Xiang, Li Xu, Huichuan Sun, Kui Wang

**Affiliations:** Department of Hepatic Surgery II, Third Affiliated Hospital of Naval Medical University (Eastern Hepatobiliary Surgery Hospital), Shanghai, People’s Republic of China; Department of Liver Surgery and Transplantation, Liver Cancer Institute and Zhongshan Hospital, Fudan University, Shanghai, People’s Republic of China; Department of Liver Surgery, Sun Yat-sen University Cancer Center, Sun Yat-sen University, Guangzhou, People’s Republic of China; Hepatobiliary Surgery Department, Guangxi Medical University Cancer Hospital, Nanning, People’s Republic of China; Department of General Surgery, Sir Run Run Shaw Hospital, College of Medicine, Institute of Minimally Invasive Surgery, Zhejiang University, Hangzhou, People’s Republic of China; Department of Hepatobiliary Surgery, First Hospital of Xian Jiaotong University, Xian, People’s Republic of China; Department of Hepatobiliary Surgery, Tianjin Medical University Cancer Institute and Hospital, Tianjin, People’s Republic of China; Department of Hepatic Surgery, Xiangya Hospital, Central South University, Hunan, People’s Republic of China; Department of General Surgery, The First Hospital of Lanzhou University, Lanzhou, People’s Republic of China; Hepatobiliary and Pancreatic Surgery, The First Affiliated Hospital of Zhengzhou University, Zhengzhou, People’s Republic of China; Department of Hepatobiliary Surgery, Mengchao Hepatobiliary Hospital of Fujian Medical University, Fuzhou, People’s Republic of China; Department of Hepatobiliary Surgery, Peking Union Hospital, Chinese Academy of Medical Sciences, Peking, People’s Republic of China; Hepatopancreatobiliary Surgery Department I, Key Laboratory of Carcinogenesis and Translational Research (Ministry of Education/Beijing), Peking, People’s Republic of China; Department of General Surgery, The First Affiliated Hospital of Dalian Medical University, Dalian, People’s Republic of China; Hepatobiliary and Pancreatic Surgery, The First Affiliated Hospital of Anhui Medical University, Hefei, People’s Republic of China; Department of General Surgery, Qilu Hospital, Shandong University, Jinan, People’s Republic of China; Institute of Hepatobiliary Surgery, Southwest Hospital, Third Military Medical University (Army Medical University), Chongqing, People’s Republic of China; Department of Hepatobiliary Surgery, Affiliated Cancer Hospital of Shandong first Medical University, Jinan, People’s Republic of China; Department of Hepatobiliary Surgery, Affiliated Cancer Hospital of Shandong first Medical University, Jinan, People’s Republic of China; Institute of Hepatobiliary Surgery, Southwest Hospital, Third Military Medical University (Army Medical University), Chongqing, People’s Republic of China; Department of General Surgery, Qilu Hospital, Shandong University, Jinan, People’s Republic of China; Hepatobiliary and Pancreatic Surgery, The First Affiliated Hospital of Anhui Medical University, Hefei, People’s Republic of China; Department of General Surgery, The First Affiliated Hospital of Dalian Medical University, Dalian, People’s Republic of China; Hepatopancreatobiliary Surgery Department I, Key Laboratory of Carcinogenesis and Translational Research (Ministry of Education/Beijing), Peking, People’s Republic of China; Department of Hepatobiliary Surgery, Peking Union Hospital, Chinese Academy of Medical Sciences, Peking, People’s Republic of China; Department of Hepatobiliary Surgery, Mengchao Hepatobiliary Hospital of Fujian Medical University, Fuzhou, People’s Republic of China; Hepatobiliary and Pancreatic Surgery, The First Affiliated Hospital of Zhengzhou University, Zhengzhou, People’s Republic of China; Department of General Surgery, The First Hospital of Lanzhou University, Lanzhou, People’s Republic of China; Department of Hepatic Surgery, Xiangya Hospital, Central South University, Hunan, People’s Republic of China; Department of Hepatobiliary Surgery, Tianjin Medical University Cancer Institute and Hospital, Tianjin, People’s Republic of China; Department of Hepatobiliary Surgery, First Hospital of Xian Jiaotong University, Xian, People’s Republic of China; Department of General Surgery, Sir Run Run Shaw Hospital, College of Medicine, Institute of Minimally Invasive Surgery, Zhejiang University, Hangzhou, People’s Republic of China; Hepatobiliary Surgery Department, Guangxi Medical University Cancer Hospital, Nanning, People’s Republic of China; Department of Liver Surgery, Sun Yat-sen University Cancer Center, Sun Yat-sen University, Guangzhou, People’s Republic of China; Department of Liver Surgery and Transplantation, Liver Cancer Institute and Zhongshan Hospital, Fudan University, Shanghai, People’s Republic of China; Department of Hepatic Surgery II, Third Affiliated Hospital of Naval Medical University (Eastern Hepatobiliary Surgery Hospital), Shanghai, People’s Republic of China

**Keywords:** hepatocellular carcinoma, conversion therapy, surgical therapy, prognosis, protective factor

## Abstract

**Background:**

The difference in the prognoses between treatment with surgical therapy and continuation of local-plus-systemic therapy following successful down-staging of intermediate-advanced hepatocellular carcinoma (HCC) remains unclear.

**Methods:**

Data of 405 patients with intermediate-advanced HCC treated at 30 hospitals across China from January 2017 to July 2022 were retrospectively reviewed. All patients received local-plus-systemic therapy and were divided into the surgical (*n* = 100) and nonsurgical groups (*n* = 305) according to whether they received surgical therapy. The differences between long-term prognoses of the 2 groups were compared. Subgroup analysis was performed in 173 HCC patients who met the criteria for surgical resection following down-staging.

**Results:**

Multivariable analysis of all patients showed that surgical therapy, hazard ratio (HR): 0.289, 95% confidence interval, CI, 0.136-0.613) was a protective factor for overall survival (OS), but not for event-free survival (EFS). Multivariable analysis of 173 intermediate-advanced HCC patients who met the criteria for surgical resection after conversion therapy showed that surgical therapy (HR: 0.282, 95% CI, 0.121-0.655) was a protective factor for OS, but not for EFS. Similar results were obtained after propensity score matching. For patients with Barcelona Clinic Liver Cancer stage B (HR: 0.171, 95% CI, 0.039-0.751) and C (HR: 0.269, 95% CI, 0.085-0.854), surgical therapy was also a protective factor for OS.

**Conclusions:**

Overall, for patients with intermediate-advanced HCC who underwent local-plus-systemic therapies, surgical therapy is a protective factor for long-term prognosis and can prolong OS, and for those who met the surgical resection criteria after conversion therapy, surgical therapy is recommended.

## Teaser

This study included 405 patients with intermediate-advanced HCC from 30 hospitals across China and investigated the difference in prognoses between treatment with surgical therapy and continuation of local-plus-systemic therapy following successful down-staging of intermediate-advanced hepatocellular carcinoma.

**Implications for Practice**

The difference in prognoses between treatment with surgical therapy and continuation of local-plus-systemic therapy following successful down-staging of intermediate-advanced hepatocellular carcinoma (HCC) is not clear. This study showed that for patients with intermediate-advanced HCC who underwent local-plus-systemic therapies, surgical therapy is a protective factor for long-term prognosis and can prolong overall survival. The same results can be obtained for intermediate-advanced HCC patients who met the criteria for surgical resection after conversion therapy. For patients who met the surgical resection criteria after conversion therapy, surgical therapy is recommended.

## Introduction

Primary hepatic carcinoma is a common malignant tumor, which has a relatively high fatality rate and ranks seventh in incidence among all malignant tumors.^1^ Hepatocellular carcinoma (HCC) is the most common type of primary hepatic carcinoma and constitutes more than 70% of primary hepatic carcinomas.^2^ China has a high incidence of liver cancer, accounting for more than 50% of new cases worldwide.^3^ Moreover, in China, HCC has already become the third largest tumor-related lethal disease.^4^ More than 60% of patients in China are diagnosed with intermediate-advanced HCC upon first diagnosis.^5,6^ Intermediate-advanced HCC patients have already lost the opportunity for radical surgery and have a relatively poor prognosis.^7,8^

Improving the prognosis of intermediate-advanced HCC patients has long been the focus of clinical research.^9,10^ With an increase in the number of targeted therapies and immunotherapies, the prognosis of HCC has significantly improved.^10,11^ Systemic therapies, such as target immunotherapy, have brought hope to intermediate-advanced HCC patients. Target immunotherapy can greatly improve the prognosis of intermediate-advanced HCC patients.^8,12^ Some patients may have a chance of radical surgery after systemic therapy to improve their prognosis.^13,14^ To further improve the overall response rate (ORR), some studies have explored a combination of systemic and local therapies for the treatment of unresectable HCC and have proposed a new surgical treatment strategy of conversion therapy.^14,15^ However, for patients who meet the criteria for surgical resection after conversion therapy, whether to undergo surgical therapy or continuation of local-plus-systemic therapy remains controversial. This study included 405 patients with intermediate-advanced HCC from 30 hospitals across China and aimed to investigate the difference in prognoses between treatment with surgical therapy and continuation of local-plus-systemic therapy following successful down-staging of intermediate-advanced hepatocellular carcinoma.

## Patients and Methods

### Patients

We retrospectively analyzed 405 intermediate-advanced HCC patients treated at 30 hospitals across China from January 2017 to July 2022. Their clinicopathological data were recorded in detail. The inclusion criteria were as follows: (1) ≥18 years old, ≤75 years old; (2) eligible HCC patients confirmed by pathological assessment or non-invasive assessment according to the American Association for the Study of Liver Diseases criteria for patients with confirmed cirrhosis^16^; (3) unresectable intermediate-advanced HCC patients (Barcelona Clinic Liver Cancer [BCLC] stage B/C) whose intrahepatic lesions could be measured; (4) Child-Pugh grade A or B7; (5) Eastern Cooperative Oncology Group score (ECOG) 0-2; (6) Tumor load was <50% of the liver volume and number of liver tumors was <10; (7) the non-surgery group continued local plus systemic therapies after receiving the initial local plus systemic therapies and did not undergo surgical therapy. The surgery group underwent surgical therapy after undergoing local plus systemic therapies and continued systemic therapies after the surgery; (8) evaluation criteria based on the Response Evaluation Criteria in Solid Tumors (RECIST) 1.1 and modified RECIST (mRECIST) criteria^17,18^; (9) complete clinicopathological data and follow-up information; and (10) treatment with target immunotherapy at 30 hospitals across China between January 2017 and December 2021. The exclusion criteria were as follows: (1) poor general condition of the patient and could not tolerate local and systemic treatment; (2) the pathological diagnosis of mixed HCC and intrahepatic cholangiocarcinoma or other non-HCC malignancies; (3) having another malignancy in the past or at the same time; (4) having an organ transplant recipient; (5) intrahepatic lesions that could not be measured; and (6) more than 2 months interval between systemic therapy and local therapy.

### Pretreatment Examination, Local Therapy, Systemic Therapy, and Surgical Therapy

A complete examination was performed after admission, including routine blood, liver, and kidney function, coagulation function, tumor markers, hepatitis markers, blood group identification, electrocardiogram, pulmonary function, gastroscopy, chest computed tomography (CT), abdominal ultrasound, and liver magnetic resonance imaging (MRI) to assess the patient’s general status.

Local therapies included transcatheter arterial chemoembolization (TACE) and hepatic arterial infusion chemotherapy (HAIC). TACE: Tumor-feeding arteries were first identified by angiography. Then, chemotherapeutic agents and iodized oil were injected into the arteries. The treatment regimen consisted of pirarubicin with lipiodol^15^. HAIC: After insertion of a microcatheter into the patient’s hepatic artery, the patient was transported to the ward to begin drug infusion. The related drug dose of hepatic arterial infusion chemotherapy was calculated based on their body surface area. Dosing regimens were as follows: oxaliplatin, 130 mg/m^2^ from hour 0-2 on day 1; leucovorin, 400 mg/m^2^ from hour 2-3 on day 1; and fluorouracil, 400 mg/m^2^ bolus at hour 3 on day 1 and 2400 mg/m^2^ over 24 hours. After drug infusion, all catheters and sheaths were removed.^19^

Systemic therapy included targeted drugs such as sorafenib, renvastinib, regofenib, and apatinib. Intermediate-advanced HCC patients were first given first-line targeted drugs, whereas those with unsatisfactory efficacy or tumor progression were switched to other drugs. All targeted drugs were administered according to the recommended dosage in the guidelines. The immunotherapy was programmed cell death protein 1 (PD-1) inhibitors, which were administered at the standard dose with a 21-day cycle.

Surgical therapy: After patients underwent local plus systemic therapies, they were assessed for whether they met the criteria for surgical resection. The resection criteria were as follows: (1) assessment of intrahepatic lesions for at least 2 months according to RECIST 1.1 or mRECIST criteria, as to whether they classified as complete response (CR)/partial response (PR)/stable disease (SD)^20^; (2) technically resectable vascular emboli; (3) achievement of R0 resection with sufficient remnant liver volume (≥40% of the standard liver volume for patients with liver cirrhosis or ≥30% of the standard liver volume for patients without liver cirrhosis)^21^; (4) no other surgical contraindications. For patients who met the criteria for surgical resection, anatomic or nonanatomic resection was performed to completely remove the tumors.

### Follow-Up, Tumor Assessment, and Study Endpoints

Patients with intermediate-advanced HCC accepted clinically evaluated for tumor response and resectability by contrast-enhanced MRI/CT and chest CT every 2 months after receiving local-plus-systemic therapy.^13^ If tumor response was assessed CR, PR, or SD according to RECIST v1.1 and modified RECIST criteria. These patients will continue to receive previous local-plus-systemic therapy, or undergo surgical therapy. Patients who had an assessment of progressive disease or intolerable side effects were switched to other therapies. Patients and their families decided whether to undergo surgical therapy if the patient met the resection criteria after conversion therapy. For those who did not chose surgical therapy, the previous local-plus-systemic therapy was continued, and patients returned to hospital for reexamination and tumor evaluation every 2 months.^13^ For those who chose to undergo surgical therapy, reexamination was performed every 2-3 months after surgical therapy, and they were evaluated for tumor recurrence. Patients who had tumor recurrences underwent therapies such as reoperation, ablation or TACE based on the specific tumor recurrence situation.

Overall survival (OS) was the primary endpoint of this study. For all patients, OS was defined as the day of the first treatment until the patient died or lost to follow up. Event-free survival (EFS) was the secondary endpoint, which was defined as the day of the first treatment until tumor recurrence (surgical group), tumor progression (nonsurgical group), death, or lost to follow up.

In the subgroup analysis, there were 173 patients who met the criteria for surgical resection after local plus systemic treatment, OS was defined as period from the day the patient met the surgical resection criteria until death or lost to follow up. EFS was defined as the period from the day the patient met the surgical resection criteria until tumor recurrence (surgical group), tumor progression (nonsurgical group), death, or lost to follow up.

### Statistical Analysis

All data in this study were analyzed using SPSS software (version 26, SPSS INC., Chicago, IL, USA) and R software version 4.0.0 (http://www.R-project.org). Medians (range) and frequencies (percentage) were used to describe measurement data and counting data, respectively, and were compared using the independent samples *t*-test, Mann-Whitney *U test*, chi-square test, or Fisher’s exact test. The OS and EFS curves were plotted using the Kaplan-Meier method, and univariate and multivariate Cox analyses were used to analyze the factors affecting OS and EFS. HR and 95% CI were used to represent the relative risks, and differences were considered statistically significant at *P* < .05.

## Results

### Patient Characteristics

**Figure 1.**
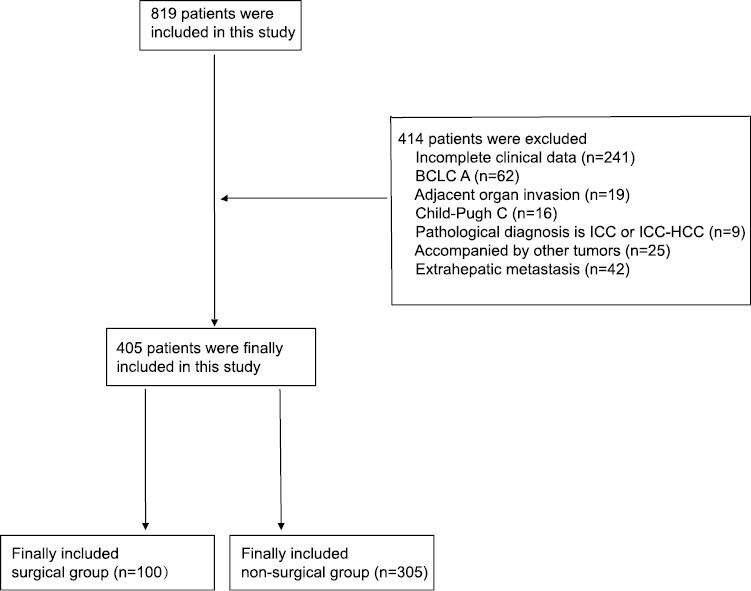
The flow chart of this study.

Fig. 1 shows the flowchart of this study. A total of 405 intermediate-advanced HCC patients were enrolled in this study. These patients were divided into the surgical (*n* = 100) or nonsurgical group (*n* = 305). The 2 groups had no significant differences in sex, age, ECOG score, preoperative antiviral therapy, alanine transaminase (ALT), alpha-fetoprotein (AFP), vitamin K absence-II (PIVKA-II), tumor diameter, number of tumors >3, type III portal vein tumor thrombus (PVTT), BCLC stage, Child-Pugh score, or local therapy (*P* > .05). The nonsurgical group had a higher HBsAg-positivity rate (92.1% vs. 83.0%), higher total bilirubin (TBIL) level (15.9 µmol/L vs. 14.5 µmol/L), lower albumin (ALB) level (38.6 g/L vs. 40.8 g/L), longer prothrombin time (PT) (12.5 s vs. 12.2 s), higher neutrophil-to-lymphocyte ratio (NLR) (2.9 vs. 2.5), and lower ORR (22.3% vs. 61.0 %) than the surgical group (Table 1).

**Table 1.** Baseline clinicopathological data in the whole group.

Variable	Number (%)/median (IQR)			*P*-value
	Non-surgical group (*n* = 305)	Surgical group (*n* = 100)	Total number (*n* = 405)	
Age, years	54.0 (48.0-60.5)	54.0 (48.0-62.0)	54.0 (48.0-61.0)	.690
ECOG score				
0/1	292 (95.7%)	94 (94.0%)	386 (95.3%)	.476
2/3	13 (4.3%)	6 (6.0%)	19 (4.7%)	
Sex				
Female	39 (12.8%)	13 (13.0%)	52 (12.8%)	.956
Male	266 (87.2%)	87 (87.0%)	353 (87.2%)	
HBsAg				
Negative	24 (7.9%)	17 (17.0%)	41 (10.1%)	.009
Positive	281 (92.1%)	83 (83.0%)	364 89.9%)	
HBV-DNA level, IU/mL				.544
<2000	154 (50.5%)	47 (47.0%)	201 (49.6)	
>2000	151 (49.5%)	53 (53.0%)	204 (50.4%)	
Antiviral therapy				.061
No	158 (51.8%)	41 (41.0%)	199 (49.1%)	
Yes	147 (48.2%)	59 (59.0%)	206 (50.9%)	
TBIL, µmol/L	15.9 (12.0-22.3)	14.5 (11.2-21.1)	15.5 (11.9-21.8)	.040
ALB, g/L	38.6 (35.5-42.0)	40.8 (37.6-44.0)	39.0 (36.0-42.4)	.001
ALT, U/L	40.0 (26.0-61.9)	38.0 (23.0-63.0)	39.0 (26.0-62.0)	.547
PT, seconds	12.5 (11.8-13.4)	12.2 (11.4-13.1)	12.4 (11.7-13.4)	.042
AFP, µg/L	574.3 (21.9-8912.0)	260.0 (13.6-5543.5)	484.0 (16.8-8359.0)	.302
PIVKA, mAU/mL	8454(448.0-14980.0)	9561.5 (177.5-9999.0)	8568.0(360.4-13218.4)	.401
NLR	2.9 (2.0-4.3)	2.5 (1.8-3.9)	2.8 (1.9-4.2)	.031
Tumor diameter, cm	8.0 (5.5-11.0)	8.6(5.9-11.3)	8.1 (5.5-11.0)	.717
Tumor number				
≤3	145 (47.5%)	56 (56.0%)	201 (49.6%)	.142
>3	160 (52.5%)	44 (44.0%)	204 (50.4%)	
PVTT				
Type I/II	258 (84.6%)	91 (91.0%)	349 (86.2%)	.107
Type III	47 (15.4%)	9 (9.0%)	56(13.8%)	
BCLC stage				
B	107 (43.4%)	36 (45.3%)	143 (35.3%)	.868
C	198 (56.6%)	64 (54.7%)	262 (64.7%)	
Child-Pugh				
A	284 (93.1%)	93 (93.0%)	377 (93.1%)	.969
B	21 (6.7%)	7 (7.0%)	28 (6.9%)	
ORR				
No	237 (77.7%)	39 (39.0%)	276 (68.1%)	<.001
Yes	68 (22.3%)	61(61.0%)	129 (31.9%)	
Local treatment				
No	101 (33.1%)	28 (28.0%)	129 (31.9%)	.341
Yes	204 (66.9%)	72 (72.0%)	276 (68.1%)	

Abbreviations: IQR: interquartile range; ECOG: Eastern Cooperative Oncology Group; HBsAg: hepatitis B surface antigen; HBV-DNA: hepatitis B virus deoxyribonucleic acid; TBIL: total bilirubin; ALB: Albumin; ALT: alanine aminotransferase; PT: prothrombin time; AFP: a-fetoprotein; PIVKA-II: Protein Induced by Vitamin K Ab; NLR: neutrophil to lymphocyte ratio; PVTT: portal vein tumor thrombus; BCLC: Barcelona Clinic Liver Cancer; ORR: Objective Response Rate.

### OS and EFS of the Whole Cohort

The 405 patients in this study had a median follow-up time of 34.3 months. The 1, 2, 3, and 4-years OS and EFS rates were 83.0%, 61.0%, 53.0%, 46% and 52.0%, 31.0%, 21.0%, 13%, respectively.

**Figure 2.**
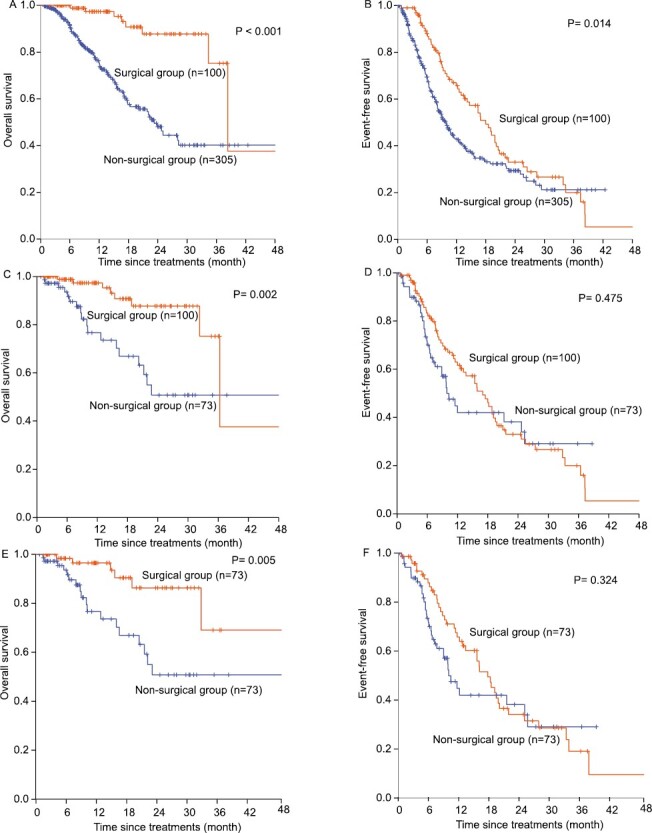
Kaplan-Meier estimate of OS and EFS for surgical group and nonsurgical group. (**A**) Kaplan-Meier estimate of OS for surgical group and nonsurgical group in the whole group. (**B**) Kaplan-Meier estimate of EFS for surgical group and nonsurgical group in the whole group. (**C**) Kaplan-Meier estimate of OS for surgical group and nonsurgical group in 173 HCC patients that met the criteria for surgical resection following down-staging. (**D**) Kaplan-Meier estimate of EFS for surgical group and nonsurgical group in 173 HCC patients that met the criteria for surgical resection following down-staging. (**E**) Kaplan-Meier estimate of OS for surgical group and non-surgical group in 146 HCC patients after PSM that met the criteria for surgical resection following down-staging. (**F**) Kaplan-Meier estimate of EFS for surgical group and non-surgical group in 146 HCC patients after PSM that met the criteria for surgical resection following down-staging.

Supplementary Table S1 shows the univariate analysis results of OS. Multivariate Cox analysis showed that PIVKA-II > 100 mAU/mL (HR: 2.661, 95% CI, 1.074-6.593), tumor number >3 (HR: 3.138, 95% CI, 2.022-4.870), type III PVTT (HR: 2.988, 95% CI, 1.807-4.941), and no ORR (HR: 2.468, 95% CI, 1.425-4.275) were independent risk factors for OS and surgical therapy (HR: 0.289, 95% CI, 0.136-0.613) and local therapy (HR: 0.560, 95% CI, 0.366-0.857) were protective factors for OS (Table 2). The 1, 2, 3, and 4-years OS rates of the surgical and nonsurgical groups were 98.0%, 88.0%, 83.0%, 62% and 78.0%, 51.0%, 41.0%, 41% (*P* < .001), respectively (Fig. 2A). The surgical and nonsurgical groups had a median OS of 38.3 months and 23.2 months, respectively.

**Table 2.** Multivariable analysis of OS and EFS of patients in the whole group.

Variable	OS			EFS		
	*P*-value	HR	95% CI	*P*-value	HR	95% CI
TBIL, µmol/L, >17	.076	1.466	0.961-2.236	-	-	-
PT, seconds, >13	.383	1.223	0.778-1.924	—	—	—
AFP, µg/L, >400	.061	1.509	0.982-2.321	—	—	—
PIVKA, mAU/mL, >100	.035	2.661	1.074-6.593	—	—	—
Surgical therapy, yes	.001	0.289	0.136-0.613	.441	0.881	0.639-1.216
Tumour number > 3	<.001	3.138	2.022-4.870	<.001	2.483	1.866-3.305
PVTT, Type III	<.001	2.988	1.807-4.941	<.001	2.549	1.793-3.623
ORR, no	.001	2.468	1.425-4.275	.004	1.568	1.153-2.131
Local treatment, yes	.008	0.560	0.366-0.857	—	—	—

Abbreviations: OS, Overall survival; EFS, Event-free survival; HR, Hazard Ratio; CI, Confiden Intenral; TBIL, total bilirubin; PT, prothrombin time; AFP, a-fetoprotein; PIVKA-II, Protein Induced by Vitamin K Ab; PVTT, portal vein tumor thrombus; ORR, Objective Response Rate.

The univariate analysis results of EFS are shown in Supplementary Table S1. Multivariate Cox analysis showed that tumor number > 3 (HR: 2.483, 95% CI, 1.866-3.305), type III PVTT (HR: 2.549, 95% CI, 1.793-3.623), and no ORR (HR: 1.568, 95% CI, 1.153-2.131) were independent risk factors for EFS (Table 2). The 1, 2, 3, and 4-years EFS rates of the surgical and nonsurgical groups were 67%, 35%, 22%, 9% and 46.0%, 31.0%, 22.0%, 22% (*P* = .014), respectively (Fig. 2B). The median EFS of the surgical and nonsurgical groups was 18.0 months and 9.9 months, respectively.

### Prognosis Analysis of Patients Who Meet the Surgical Resection Criteria After Local Plus Systemic Treatment

Of the 405 HCC patients, 173 patients met the criteria for surgical resection after local-plus-systemic therapy, among which 100 patients eventually received surgical therapy and 73 continued local-plus-systemic therapy. The 173 HCC patients who met the criteria for surgical resection had a median OS of 36.3 months and a median EFS of 15.4 months.

The clinicopathological data of the 173 HCC patients are listed in Supplementary Table S2. There was no significant difference between the surgical and nonsurgical groups in terms of sex, age, ECOG score, preoperative antiviral therapy, TBIL, ALB, ALT, PT, AFP, PIVKA-II, tumor diameter, number of tumors >3, type III PVTT, BCLC stage, Child-Pugh score, and local treatment (*P* > .05). The nonsurgical group had a higher HBsAg-positivity rate (93.2% vs. 83.0%), higher NLR (3.3 vs. 2.5), and lower ORR (61.0 % vs. 86.3%) than the surgical group.

Supplementary Table S3 summarizes the univariate analysis results of OS. Multivariate Cox analysis showed that tumor number > 3 (HR: 4.518, 95% CI, 1.890-10.802) was an independent risk factor for OS and that antiviral therapy (HR: 0.356, 95% CI, 0.157-0.806) and surgical therapy (HR: 0.282, 95% CI, 0.121-0.655) were protective factors for OS (Table 3). The 1, 2, 3, and 4-years OS rates of the surgical and nonsurgical groups were 97%, 87%, 81%, 54% and 80%, 54%, 54%, 54% (*P* = .002), respectively (Fig. 2C). The median OS was 36.3 months for the surgical group and not available for the nonsurgical group. The univariate analysis results of EFS are listed in Supplementary Table S3. Multivariate Cox analysis showed that tumor number >3 (HR: 2.465, 95% CI, 1.591-3.817) and type III PVTT (HR: 2.483, 95% CI, 1.367-4.511) were independent risk factors for EFS (Table 3). The 1, 2, 3, and 4-year EFS rates of the surgical and nonsurgical groups were 64%, 35%, 22%, 7% and 47%, 43%, 29%, 29% (*P* = .475), respectively (Fig. 2D). The median EFS of the surgical and non-surgical groups was 17.0 and 9.9 months, respectively.

**Table 3.** Multivariable analysis of OS and EFS of patients who meet the surgical resection criteria after local plus systemic treatment.

Variable	OS			EFS		
	*P*-value	HR	95% CI	*P*-value	HR	95% CI
Before PSM						
Antiviral therapy, yes	.013	0.356	0.157-0.806	—	—	—
Surgical therapy, yes	.003	0.282	0.121-0.655	—	—	—
Tumor number > 3	.001	4.518	1.890-10.802	<.001	2.465	1.591-3.817
PVTT, Type III	—	—	—	.003	2.483	1.367-4.511
After PSM						
Antiviral therapy, yes	.144	0.511	0.208-1.256	—	—	—
Surgical therapy, yes	.002	0.220	0.082-0.586	—	—	—
Tumour number > 3	<.001	5.408	2.196-13.317	<.001	2.373	1.472-3.827
PVTT, Type III	—	—	—	.010	2.285	1.222-4.275
ORR, no	.018	3.348	1.231-9.105	—	—	—

Abbreviations: OS: overall survival; EFS: event-free survival; HR: hazard ratio; CI: confidence internal; PVTT: portal vein tumor thrombus.

After PSM, 146 patients from the nonsurgical group (*n* = 73) and surgical group (n = 73) were selected for further analysis. There was no significant difference in all clinicopathological features (Supplementary Table S4). Supplementary Table S5 lists the univariate analysis results of OS. Multivariate Cox analysis showed that tumor number >3 (HR: 5.408, 95% CI, 2.196-13.317) and no ORR (HR: 3.348, 95% CI, 1.231-9.105) were independent risk factors for OS and that surgical therapy (HR: 0.220, 95% CI, 0.082-0.586) was a protective factor for OS (Table 3). The 1, 2, 3, and 4-years OS rates of the surgical and non-surgical groups were 96%, 86%, 78%, 78% and 80%, 54%, 54%, 54% (*P* = .005), respectively (Fig. 2E). The univariate analysis results of EFS are summarized in Supplementary Table S5. Multivariate Cox analysis showed that tumor number >3 (HR: 2.373, 95% CI, 1.472-3.827) and type III PVTT (HR: 2.285, 95% CI, 1.222-4.275) were independent risk factors for EFS (Table 3). The 1, 2, 3, and 4-year EFS rates of the surgical and nonsurgical groups were 66%, 37%, 23%, 14% and 47%, 43%, 29%, 29% (*P* = .324), respectively (Fig. 2F).

### Prognosis Analysis of Patients According to the BCLC Stage Who Meet The Surgical Resection Criteria After Local-Plus-Systemic Therapy

Among the 173 HCC patients, 61 were in stage B, and 112 were in stage C. Among BCLC stage B patients, there were 36 and 25 patients in the surgical and nonsurgical groups, respectively.

**Figure 3.**
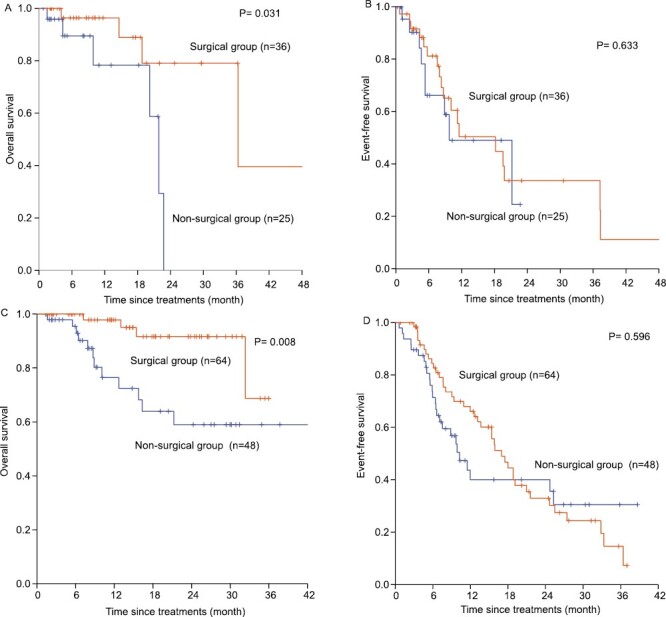
Kaplan-Meier estimate of OS and EFS for surgical group and non-surgical group for 173 HCC patients that met the criteria for surgical resection following down-staging according to BCLC stages B and C. (**A**) Kaplan-Meier estimate of OS for surgical group and non-surgical group for HCC patients that met the criteria for surgical resection following down-staging according to BCLC stage B. (**B**) Kaplan-Meier estimate of EFS for surgical group and non-surgical group for HCC patients that met the criteria for surgical resection following down-staging according to BCLC stage B. (**C**) Kaplan-Meier estimate of OS for surgical group and nonsurgical group for HCC patients that met the criteria for surgical resection following down-staging according to BCLC stage C. (**D**) Kaplan-Meier estimate of EFS for surgical group and non-surgical group for HCC patients that met the criteria for surgical resection following down-staging according to BCLC stage C.

Supplementary Table S6 shows the univariate analysis results of OS for BCLC stage B patients. Multivariate Cox analysis showed that surgical therapy (HR: 0.171, 95% CI, 0.039-0.751) was a protective factor and tumor number >3 (HR: 5.872, 95% CI, 1.204-28.632) was an independent risk factor for OS (Supplementary Table S7). The 1, 2, 3, and 4-years OS rates of the surgical and nonsurgical groups were 96%, 78%, 78%, 47% and 82%, 27%, 0, 0 (*P* = .031), respectively (Fig. 3A). The median OS of the surgical and nonsurgical groups were 36.3 and 21.9 months, respectively.

The univariate analysis results of EFS for BCLC stage B patients are summarized in Supplementary Table S6. Multivariate Cox analysis showed that cirrhosis (HR: 2.400, 95% CI, 1.068-5.391) was an independent risk factor for EFS (Supplementary Table S7). The 1, 2, 3, and 4-years EFS rates of the surgical and nonsurgical groups were 56%, 36%, 36%, 12%, and 57%, 34%, 0, 0 (*P* = .633), respectively (Fig. 3B). The median EFS of the surgical and nonsurgical groups was 18.2 and 9.7 months, respectively.

For BCLC stage C patients, there were 64 patients in the surgical group and 48 in the nonsurgical group. Supplementary Table S8 lists the univariate analysis results of OS. Multivariate Cox analysis showed that surgical therapy (HR: 0.269, 95% CI, 0.085-0.854) was a protective factor and that tumor number >3 (HR: 3.788, 95% CI, 1.301-11.030) was an independent risk factor for OS (Supplementary Table S9). The 1, 2, 3, and 4-years OS rates of the surgical and nonsurgical groups were 98%, 91%, 81%, 81% and 79%, 61%, 61%, 61% (*P* = .008), respectively (Fig. 3C). The median OS was not available for either the surgical or nonsurgical group. The univariate analysis results of EFS for BCLC stage C patients are summarized in Supplementary Table S8. Multivariate Cox analysis showed that NLR > 2.15 (HR: 0.434, 95% CI, 0.251-0.751) was a protective factor and that type III PVTT (HR: 2.965, 95% CI, 1.504-5.845) and tumor number >3 (HR: 2.709, 95% CI, 1.576-4.657) were independent risk factors for EFS (Supplementary Table S9). The 1, 2, 3, and 4-years EFS rates of the surgical and nonsurgical groups were 68%, 36%, 18%, 6% and 42%, 42%, 28%, 28% (*P* = .596), respectively (Fig. 3D), while the median EFS was 17.0 and 10.3 months for the surgical and nonsurgical groups, respectively.

### Analysis of Tumor Recurrence or Progression

Among the 405 HCC patients, there were 222 patients with tumor recurrence or progression. There were 58 of 100 patients with tumor recurrence in the surgical group and 164 of 305 patients with tumor progression in the nonsurgical group. There were 43 patients who had intrahepatic recurrence, 9 had extrahepatic recurrence, and 6 had intra-plus extrahepatic recurrence in the surgical group, while there were 83 patients who had intrahepatic progression, 45 had extrahepatic progression, and 36 had intra-plus extrahepatic progression in the non-surgical group, with statistically significant differences (*P* = .008). Regarding the sites of tumor recurrence or progression, in the surgical group, there were 50 patients with intrahepatic recurrences, 10 with pulmonary metastases, 4 with abdominal lymph node metastases, and 1 with recurrence at another site. In the non-surgical group, there were 119 patients with intrahepatic progression, 68 with pulmonary metastases, 8 with abdominal lymph node metastases, 2 with bone metastases, and 5 with recurrences at other sites (Table 4).

**Table 4.** Patterns of tumor recurrence or progression in the whole group.

Parameter	Resection group (*n* = 100)	No resection group (*n* = 305)	*P*-value
Total tumor recurrence or progression, *n*	58^*^	164^†^	.461
Types of recurrence or progression, *n*			
Intrahepatic only	43 (74.1%)	83 (50.6%)	.008
Extrahepatic only	9 (15.5%)	45 (27.4%)	
Intrahepatic plus extrahepatic	6 (10.4%)	36 (22.0%)	

^*^58 patients had tumor recurrences.

^†^164 patients had tumor progression.

## Discussion

To our knowledge, this study is the first multicenter large sample conversion therapy study conducted in China that enrolled 406 intermediate-advanced HCC patients from 30 hospitals. It is also the first study to compare the difference in prognoses between treatment with surgical therapy and continuation of local-plus-systemic therapy following successful down-staging of intermediate-advanced HCC. The surgical and nonsurgical groups had median OS of 38.3 months and 23.2 months, respectively, and the median OS of the non-surgical patients was consistent with that described previously.^22,23^ The 1, 2, 3, and 4-years OS rates of surgical and nonsurgical groups were 98.0%, 88.0%, 83.0%, 62%, and 78.0%, 51.0%, 41.0%, 41.0%, respectively (*P* < .001). Therefore, for intermediate-advanced HCC patients who received local-plus-systemic therapy initially and successful down-staging to meet the criteria for surgical resection, surgical therapy could improve patient’s OS. The surgical group had a median OS of 38.3 months, which was significantly better than that of the nonsurgical group. It was also significantly better than the prognosis of intermediate-advanced HCC patients who received nonsurgical therapy in previous studies.^24-26^ Our study also found that surgical therapy was not a protective factor for EFS. The 1, 2, 3, and 4-years EFS rates of the surgical group were 67%, 35%, 22%, and 9%, respectively, with a median EFS of 18.0 months. These results indicate that for intermediate-advanced HCC, tumor recurrence remains common even for patients with successful down-staging and surgical resection, which is consistent with the results of a previous study.^27^ Therefore, for intermediate-advanced HCC patients who underwent surgical resection following successful down-staging, postoperative treatment is still necessary.

We further analyzed the 173 intermediate-advanced HCC patients who met the surgical resection criteria after local plus systemic treatment. For intermediate-advanced HCC patients who met the criteria for surgical resection after local-plus-systemic therapy, the 1, 2, 3, and 4-years OS rates of the surgical and nonsurgical groups were 97%, 87%, 81%, 54% and 80%, 54%, 54%, 54% (*P* = .002), respectively. This means that for these patients, surgical therapy could improve their OS. There are differences in multiple variables between the 2 groups, including the ORR rate. Is the difference in prognosis due to differences in ORR rates? In order to balance the differences between the 2 groups of 173 HCC patients due to factors such as ORR, we conducted a PSM analysis. Similar results were obtained after PSM. Furthermore, for patients of BCLC stages B and C, the prognosis of the surgical group was better than that of the non-surgical group. A previous study also reported that for BCLC stage B/C patients, the surgical group had a better survival than that receiving local therapy only.^28^ However, our results demonstrate that the 1, 2, 3, and 4-years EFS rates of the surgical and nonsurgical groups were 64%, 35%, 22%, 7% and 47%, 43%, 29%, 29% (*P* = .475), respectively. The median EFS of the surgical and non-surgical groups was 17.0 and 9.9 months, respectively. These results suggest that surgical therapy is not a protective factor for EFS. In other words, for intermediate-advanced HCC patients who meet the criteria for surgical resection after local plus systemic treatment, surgical therapy cannot improve the patients’ EFS, but can improve their OS. We attribute this to the fact that although surgical therapy cannot improve the EFS of patients, it can change the tumor recurrence pattern, tumor progression pattern, and tumor load of patients. Thus, even if tumor recurs, patients of the surgical group have fewer recurrent lesions and a lower tumor load than those of the nonsurgical group. We further compared the tumor recurrence pattern and tumor progression pattern between the 2 groups. Although the rate of tumor recurrence was not significantly different (*P* = .461), the surgical group had a significantly higher rate of intrahepatic recurrence and a significantly lower rate of extrahepatic recurrence or progression than the nonsurgical group (*P* = .008). The finding that tumor recurrence is mostly intrahepatic after surgical therapy is consistent with that of a previous study.^29^ Some patients with intrahepatic recurrence after surgical therapy can still receive radical treatment such as ablation. Patients with extrahepatic recurrence or metastasis, especially with multiple extrahepatic recurrences or metastases, have a relatively poor prognosis.^30^ In addition, surgical therapy can decrease the tumor load after tumor recurrence. Thus, even if the tumor recurs, patients with tumor recurrence after surgical therapy have a lower tumor load than those in the nonsurgical group. Consequently, although patients who received surgical therapy after conversion did not show significantly improved EFS compared with non-surgical patients, their OS could still be improved.

The limitation of this study is that it was a retrospective study. Besides, most patients are HBsAg positive in this study. As HBsAg positive HCC patients often have a more favorable course than HBsAg negative HCC. In other words, it is unclear whether the results of this study are related to HBV infection. Therefore, prospective studies with a large sample size are thus required to further validate our results.

## Conclusion

The prognosis of intermediate-advanced HCC has improved greatly because of local-plus-systemic therapy. Surgical therapy is still an important treatment for improving the long-term prognosis of these patients. For intermediate-advanced HCC patients who meet the criteria for surgical resection after successful down-staging using local and systemic therapies, surgical therapy can still significantly improve the patient’s long-term prognosis compared with non-surgical therapy. For these patients, surgical therapy should be recommended.

## Supplementary Material

Supplementary material is available at *The Oncologist* online.

oyad277_suppl_Supplementary_Table_S1

oyad277_suppl_Supplementary_Table_S2

oyad277_suppl_Supplementary_Table_S3

oyad277_suppl_Supplementary_Table_S4

oyad277_suppl_Supplementary_Table_S5

oyad277_suppl_Supplementary_Table_S6

oyad277_suppl_Supplementary_Table_S7

oyad277_suppl_Supplementary_Table_S8

oyad277_suppl_Supplementary_Table_S9

## Data Availability

The data supporting the findings of this study are available upon request from the corresponding author. The data are not publicly available due to privacy or ethical restrictions.
