# Supplementary material for: Prognoses of Patients Treated With Surgical Therapy Versus Continuation of Local-Plus-Systemic Therapy Following Successful Down-Staging of Intermediate-Advanced Hepatocellular Carcinoma: A Multicenter Real-World Study
Source: Oncologist. 2023 Oct 24;29(4):e487–97. doi: 10.1093/oncolo/oyad277 (PMC10994252; doi:10.1093/oncolo/oyad277)
Supplement: oyad277_suppl_Supplementary_Table_S1 [file oyad277_suppl_supplementary_table_s1.docx]

| **Supplement Table 1. Univarate analysis of OS and EFS of patients in the whole group** | | | | | | |
| --- | --- | --- | --- | --- | --- | --- |
| **Variable** | **OS** | | | **EFS** | | |
|  | ***P-value*** | **HR** | **95%CI** | ***P-value*** | **HR** | **95%CI** |
| Age, years, >60 | .842 | 1.049 | 0.656-1.677 | .946 | 0.989 | 0.723-1.354 |
| Sex, male | .442 | 0.801 | 0.454-1.412 | .875 | 1.033 | 0.686-1.557 |
| ECOG score, 2/3 | .440 | 1.314 | 0.657-2.626 | .912 | 1030 | 0.607-1.748 |
| HBsAg, positive | .564 | 1.238 | 0.599-2.555 | .227 | 1.328 | 0.838-2.104 |
| HBV-DNA, IU/mL, > 2000 | .885 | 1.030 | 0.690-1.536 | .941 | 0.990 | 0.760-1.290 |
| Antiviral therapy, yes | .064 | 0.681 | 0.454-1.022 | .090 | 1.257 | 0.965-1.637 |
| NLR >2.15 | .539 | 1.149 | 0.738-1.789 | .638 | 0.935 | 0.706-1.238 |
| TBIL, µmol/L, >17 | .007 | 1.736 | 1.159-2.600 | .213 | 1.188 | 0.906-1.557 |
| ALB, g/L, ≥35 | .751 | 1.099 | 0.612-1.974 | .181 | 1.301 | 0.885-1.912 |
| ALT, U/L, >80 | .668 | 1.091 | 0.732-1.626 | .724 | 1.049 | 0.806-1.365 |
| PT, seconds, >13 | .045 | 1.539 | 1.010-2.346 | .221 | 1.194 | 0.899-1.585 |
| AFP, µg/L, >400 | .027 | 1.575 | 1.052-2.359 | .195 | 1.190 | 0.914-1.549 |
| PIVKA, mAU/mL, >100 | .015 | 3.062 | 1.244-7.538 | .966 | 1.008 | 0.707-1.436 |
| Surgical therapy, yes | <.001 | 0.187 | 0.090-0.385 | .014 | 0.684 | 0.506-0.925 |
| Cirrhosis, yes | .666 | 1.095 | 0.726-1.650 | .073 | 1.281 | 0.978-1.678 |
| Tumour size ≥ 5 cm | .894 | 0.963 | 0.555-1.673 | .769 | 0.949 | 0.669-1.347 |
| Tumour number >3 | <.001 | 2.696 | 1.767-4.112 | <.001 | 2.314 | 1.750-3.059 |
| PVTT, Type III | <.001 | 2.443 | 1.505-3.966 | <.001 | 2.152 | 1.530-3.026 |
| ORR, no | <.001 | 3.342 | 1.973-5.659 | <.001 | 1.695 | 1.269-2.264 |
| Local treatment, yes | .006 | 0.571 | 0.382-0.854 | .307 | 0.865 | 0.656-1.142 |
| **Abbreviation:** OS, Overall survival; EFS, Event-free survival; HR, Hazard Ratio; CI, Confiden Intenral; ECOG, Eastern Cooperative Oncology Group; HBsAg, hepatitis B surface antigen; HBV-DNA, hepatitis B virus deoxyribonucleic acid; TBIL, total bilirubin; ALB, Albumin; ALT, alanine aminotransferase; PT, prothrombin time; AFP, a-fetoprotein; PIVKA-II, Protein Induced by Vitamin K Ab; NLR, neutrophil to lymphocyte ratio; PVTT, portal vein tumor thrombus; ORR, Objective Response Rate. | | | | | | |
