# Supplementary material for: Prognoses of Patients Treated With Surgical Therapy Versus Continuation of Local-Plus-Systemic Therapy Following Successful Down-Staging of Intermediate-Advanced Hepatocellular Carcinoma: A Multicenter Real-World Study
Source: Oncologist. 2023 Oct 24;29(4):e487–97. doi: 10.1093/oncolo/oyad277 (PMC10994252; doi:10.1093/oncolo/oyad277)
Supplement: oyad277_suppl_Supplementary_Table_S2 [file oyad277_suppl_supplementary_table_s2.docx]

| **Supplement Table 2. Baseline clinicopathological data of patients who meet the surgical resection criteria after local plus systemic treatment** | | | | |
| --- | --- | --- | --- | --- |
| **Variable** | **Number (%)/median (IQR)** | | | ***P-value*** |
|  | **Non-surgical group**  **(n = 73)** | **Surgical group**  **(n = 100)** | **Total number**  **(n = 173)** |  |
| Age, years | 53.0 (48.0-58.0) | 54.0 (48.0-62.0) | 54.0 (48.0-60.0) | .587 |
| ECOG score |  |  |  |  |
| 0/1 | 68 (93.2%) | 94 (94.0%) | 162 (93.6%) | 1.000 |
| 2/3 | 5 (6.8%) | 6 (6.0%) | 11 (6.4%) |  |
| Sex |  |  |  |  |
| Female | 7 (9.6%) | 13 (13.0%) | 20 (11.6%) | .488 |
| Male | 66 (90.4%) | 87 (87.0%) | 153 (88.4%) |  |
| HBsAg |  |  |  |  |
| Negative | 5 (6.8%) | 17 (17.0%) | 22 (12.7%) | .048 |
| Positive | 68 (93.2%) | 83 (83.0%) | 151 (87.3%) |  |
| HBVDNA, IU/mL |  |  |  |  |
| ≤ 2000 | 34 (46.6%) | 47 (47.0%) | 81 (46.8%) | .956 |
| > 2000 | 39 (53.4%) | 53 (53.0%) | 92 (53.2%) |  |
| Antiviral therapy, |  |  |  |  |
| No | 36 (49.3%) | 41 (41.0%) | 77 (44.5%) | .277 |
| Yes | 37 (50.7%) | 59 (59.0%) | 96 (55.5%) |  |
| TBIL, µmol/L | 14.0 (11.9-22.3) | 14.5 (11.2-21.1) | 14.2 (11.4-21.4) | .319 |
| ALB, g/L | 39.3 (36.1-42.2) | 40.8 (37.6-44.0) | 40.0 (37.0-43.5) | .097 |
| ALT, U/L | 37.0 (25.1-57.8) | 38.0 (23.0-63.0) | 38.0 (25.0-59.0) | .956 |
| PT, seconds | 12.4 (11.8-13.2) | 12.2 (11.4-13.1) | 12.3 (11.6-13.1) | .177 |
| AFP, µg/L | 334.5 (20.2-7178.7) | 260.0 (13.6-5543.5) | 316.7 (14.0-6785.4) | .674 |
| PIVKA, mAU/mL | 3700.1(227.5-9999.0) | 9561.5 (177.5-9999.0) | 6692.0(201.0-9999.0) | .834 |
| NLR | 3.3 (2.2-5.3) | 2.5 (1.8-3.9) | 2.8 (1.9-4.4) | .010 |
| Tumour diameter, cm | 9.0 (4.3-11.4) | 8.6(5.9-11.3) | 8.6(5.5-11.2) | .784 |
| Tumor number |  |  |  |  |
| ≤3 | 37 (50.7%) | 56 (56.0%) | 93 (53.8%) | .489 |
| >3 | 36 (49.3%) | 44 (44.0%) | 80 (46.2%) |  |
| PVTT |  |  |  |  |
| Type I/II | 61 (83.6%) | 91 (91.0%) | 152 (87.9%) | .139 |
| Type III | 12 (16.4%) | 9 (9.0%) | 21 (12.1%) |  |
| BCLC |  |  |  |  |
| B | 25(34.2%) | 36 (36.0%) | 61 (35.3%) | .812 |
| C | 48 (65.8%) | 64 (64.0%) | 112 (64.7%) |  |
| Child Pugh |  |  |  |  |
| A | 65(89.0%) | 93 (93.0%) | 158 (91.3%) | .361 |
| B | 8 (11.0%) | 7 (7.0%) | 15 (8.7%) |  |
| ORR |  |  |  |  |
| No | 10 (13.7%) | 39 (39.0%) | 49 (28.3%) | <.001 |
| Yes | 63 (86.3%) | 61(61.0%) | 124 (71.7%) |  |
| Local treatment |  |  |  |  |
| No | 23 (31.5%) | 28 (28.0%) | 51 (29.5%) | .617 |
| Yes | 50 (68.5%) | 72 (72.0%) | 122 (70.5%) |  |
| **Abbreviation:** IQR, interquartile range; ECOG, Eastern Cooperative Oncology Group, HBsAg, hepatitis B surface antigen; HBV-DNA, hepatitis B virus deoxyribonucleic acid; TBIL, total bilirubin; ALB, Albumin; ALT, alanine aminotransferase; PT, prothrombin time; AFP, a-fetoprotein; PIVKA-II, Protein Induced by Vitamin K Ab; NLR, neutrophil to lymphocyte ratio; PVTT, portal vein tumor thrombus; BCLC, Barcelona Clinic Liver Cancer; ORR, Objective Response Rate. | | | | |
