# Supplementary material for: Prognoses of Patients Treated With Surgical Therapy Versus Continuation of Local-Plus-Systemic Therapy Following Successful Down-Staging of Intermediate-Advanced Hepatocellular Carcinoma: A Multicenter Real-World Study
Source: Oncologist. 2023 Oct 24;29(4):e487–97. doi: 10.1093/oncolo/oyad277 (PMC10994252; doi:10.1093/oncolo/oyad277)
Supplement: oyad277_suppl_Supplementary_Table_S3 [file oyad277_suppl_supplementary_table_s3.docx]

| **Supplement Table 3. Univarate analysis of OS and EFS of patients who meet the surgical resection criteria after local plus systemic treatment** | | | | | | |
| --- | --- | --- | --- | --- | --- | --- |
| **Variable** | **OS** | | | **EFS** | | |
|  | ***P-value*** | **HR** | **95%CI** | ***P-value*** | **HR** | **95%CI** |
| Age, years, >60 | .408 | 1.447 | 0.603-3.471 | .626 | 1.132 | 0.687-1.864 |
| Sex, male | .886 | 0.915 | 0.273-3.072 | .709 | 0.882 | 0.457-1.704 |
| ECOG score, 3/4 | .733 | 1.237 | 0.365-4.196 | .673 | 0.853 | 0.409-1.780 |
| HBsAg, positive | .605 | 0.754 | 0.258-2.198 | .731 | 1.117 | 0.594-2.100 |
| HBV-DNA, IU/mL, > 2000 | .946 | 0.974 | 0.448-2.118 | .629 | 1.107 | 0.733-1.672 |
| Antiviral therapy, yes | .014 | 0.361 | 0.160-0.811 | .067 | 1.493 | 0.972-2.294 |
| NLR >2.15 | .333 | 1.585 | 0.624-4.029 | .557 | 0.879 | 0.571-1.352 |
| TBIL, µmol/L, >17 | .288 | 1.544 | 0.692-3.445 | .269 | 1.276 | 0.828-1.968 |
| ALB, g/L, ≥35 | .397 | 0.629 | 0.215-1.838 | .358 | 0.758 | 0.421-1.367 |
| ALT, U/L, >80 | .940 | 1.030 | 0.474-2.237 | .851 | 0.962 | 0.638-1.449 |
| PT, seconds, >13 | .792 | 1.131 | 0.453-2.825 | .451 | 1.196 | 0.751-1.906 |
| AFP, µg/L, >400 | .308 | 0.663 | 0.300-1.461 | .284 | 0.799 | 0.529-1.205 |
| PIVKA, mAU/mL, >100 | .724 | 1.243 | 0.371-4.159 | .303 | 0.762 | 0.453-1.279 |
| Surgical therapy, yes | .002 | 0.264 | 0.115-0.610 | .475 | 0.857 | 0.562-1.308 |
| Cirrhosis, yes | .887 | 1.061 | 0.468-2.409 | .166 | 1.348 | 0.884-2.057 |
| Tumour size ≥ 5 cm | .433 | 0.692 | 0.276-1.736 | .451 | 0.828 | 0.508-1.351 |
| Tumour number >3 | <.001 | 4.821 | 2.054-11.320 | <.001 | 2.315 | 1.500-3.572 |
| PVTT, Type III | .246 | 1.890 | 0.644-5.541 | .011 | 2.152 | 1.191-3.888 |
| ORR, no | .298 | 1.537 | 0.684-3.451 | .336 | 1.248 | 0.795-1.957 |
| Local treatment, yes | .131 | 0.543 | 0.246-1.199 | .545 | 1.149 | 0.733-1.802 |
| **Abbreviation:** OS, Overall survival; EFS, Event-free survival; HR, Hazard Ratio; CI, Confiden Intenral; ECOG, Eastern Cooperative Oncology Group; HBsAg, hepatitis B surface antigen; HBV-DNA, hepatitis B virus deoxyribonucleic acid; TBIL, total bilirubin; ALB, Albumin; ALT, alanine aminotransferase; PT, prothrombin time; AFP, a-fetoprotein; PIVKA-II, Protein Induced by Vitamin K Ab; NLR, neutrophil to lymphocyte ratio; PVTT, portal vein tumor thrombus; ORR, Objective Response Rate. | | | | | | |
