# Supplementary material for: Prognoses of Patients Treated With Surgical Therapy Versus Continuation of Local-Plus-Systemic Therapy Following Successful Down-Staging of Intermediate-Advanced Hepatocellular Carcinoma: A Multicenter Real-World Study
Source: Oncologist. 2023 Oct 24;29(4):e487–97. doi: 10.1093/oncolo/oyad277 (PMC10994252; doi:10.1093/oncolo/oyad277)
Supplement: oyad277_suppl_Supplementary_Table_S4 [file oyad277_suppl_supplementary_table_s4.docx]

| **Supplement Table 4. Baseline clinicopathological data of patients who meet the surgical resection criteria after local plus systemic treatment after PSM** | | | | |
| --- | --- | --- | --- | --- |
| **Variable** | **Number (%)/median (IQR)** | | | ***P-value*** |
|  | **Non-surgical group**  **(n = 73)** | **Surgical group**  **(n = 73)** | **Total number**  **(n = 146)** |  |
| Age, years | 53.0(48.0-58.0) | 53.5 (47.3-59.8) | 53.0 (48.0-58.0) | .877 |
| ECOG score |  |  |  |  |
| 0/1 | 68 (93.2%) | 68 (93.2%) | 136 (93.2%) | 1.000 |
| 2/3 | 5 (6.8%) | 5 (6.8%) | 10 (6.8%) |  |
| Sex |  |  |  |  |
| Female | 7 (9.6%) | 8 (11.0%) | 15 (10.3%) | .785 |
| Male | 66 (90.4%) | 65 (89.0%) | 131 (89.7%) |  |
| HBsAg |  |  |  |  |
| Negative | 5 (6.8%) | 9 (12.3%) | 14 (19.6%) | .261 |
| Positive | 68 (93.2%) | 64 (87.7%) | 132 (90.4%) |  |
| HBVDNA, IU/mL |  |  |  |  |
| ≤ 2000 | 34 (46.6%) | 35 (47.9%) | 69 (47.3%) | .868 |
| > 2000 | 39 (53.4%) | 38 (52.1%) | 77 (52.7%) |  |
| Antiviral therapy, |  |  |  |  |
| No | 36 (49.3%) | 27 (37.0%) | 63 (43.2%) | .133 |
| Yes | 37 (50.7%) | 46 (63.0%) | 83 (56.8%) |  |
| TBIL, µmol/L | 14.0 (11.9-22.3) | 14.8 (11.3-21.4) | 14.3 (11.6-21.6) | .442 |
| ALB, g/L | 39.3 (36.1-42.2) | 40.7 (37.5-3.5) | 40.0 (36.7-43.4) | .192 |
| ALT, U/L | 37.0 (25.1-57.8) | 39.7 (23.0-64.0) | 39.0 (25.0-59.5) | .647 |
| PT, seconds | 12.4 (11.8-13.2) | 12.4 (11.4-13.1) | 12.4 (11.6-13.2) | .359 |
| AFP, µg/L | 334.5(20.2-7178.7) | 361.1 (14.3-3905.5) | 361.0 (15.7-6731.7) | .600 |
| PIVKA, mAU/mL | 3700.1(227.5-9999.0) | 9999.0(203.1-21936.8) | 6109.0(227.5-10374.0) | .462 |
| NLR | 3.3 (2.2-5.3) | 2.9 (2.1-4.5) | 3.1 (2.1-4.7) | .577 |
| Tumour diameter, cm | 9.0 (4.3-11.4) | 9.0(6.0 -11.8) | 9.0(5.4-11.7) | .459 |
| Tumor number |  |  |  |  |
| ≤3 | 37 (50.7%) | 41 (56.2%) | 78 (53.4%) | .507 |
| >3 | 36 (49.3%) | 32 (43.8%) | 68 (46.6%) |  |
| PVTT |  |  |  |  |
| Type I/II | 61 (83.6%) | 66 (90.4%) | 127 (87.0%) | .219 |
| Type III | 12 (16.4%) | 7 (9.6%) | 19 (13.0%) |  |
| BCLC |  |  |  |  |
| B | 25(34.2%) | 28 (38.4%) | 53 (36.3%) | .606 |
| C | 48 (65.8%) | 45 (61.6%) | 93 (63.7%) |  |
| Child Pugh |  |  |  |  |
| A | 65(89.0%) | 69 (94.5%) | 134 (91.8 %) | .228 |
| B | 8 (11.0%) | 4(5.5%) | 12 (8.2%) |  |
| ORR |  |  |  |  |
| No | 10 (13.7%) | 15 (20.5%) | 25 (17.1%) | .272 |
| Yes | 63 (86.3%) | 61(61.0%) | 124 (71.7%) |  |
| Local treatment |  |  |  |  |
| No | 23 (31.5%) | 23 (31.5%) | 46 (29.5%) | 1.000 |
| Yes | 50 (68.5%) | 50 (68.5%) | 100 (68.5%) |  |
| **Abbreviation:** IQR, interquartile range; ECOG, Eastern Cooperative Oncology Group, HBsAg, hepatitis B surface antigen; HBV-DNA, hepatitis B virus deoxyribonucleic acid; TBIL, total bilirubin; ALB, Albumin; ALT, alanine aminotransferase; PT, prothrombin time; AFP, a-fetoprotein; PIVKA-II, Protein Induced by Vitamin K Ab; NLR, neutrophil to lymphocyte ratio; PVTT, portal vein tumor thrombus; BCLC, Barcelona Clinic Liver Cancer; ORR, Objective Response Rate. | | | | |
