# Supplementary material for: Prognoses of Patients Treated With Surgical Therapy Versus Continuation of Local-Plus-Systemic Therapy Following Successful Down-Staging of Intermediate-Advanced Hepatocellular Carcinoma: A Multicenter Real-World Study
Source: Oncologist. 2023 Oct 24;29(4):e487–97. doi: 10.1093/oncolo/oyad277 (PMC10994252; doi:10.1093/oncolo/oyad277)
Supplement: oyad277_suppl_Supplementary_Table_S5 [file oyad277_suppl_supplementary_table_s5.docx]

| **Supplement Table 5. Univarate analysis of OS and EFS of patients who meet the surgical resection criteria after local plus systemic treatment after PSM** | | | | | | |
| --- | --- | --- | --- | --- | --- | --- |
| **Variable** | **OS** | | | **EFS** | | |
|  | ***P-value*** | **HR** | **95%CI** | ***P-value*** | **HR** | **95%CI** |
| Age, years, >60 | .343 | 1.533 | 0.634-3.706 | .634 | 1.147 | 0.651-2.022 |
| Sex, male | .508 | 0.663 | 0.1972.234 | .814 | 0.904 | 0.392-2.088 |
| ECOG score, 3/4 | .901 | 1.081 | 0.315-3.706 | .566 | 0.793 | 0.359-1.752 |
| HBsAg, positive | .612 | 0.730 | 0.216-2.461 | .426 | 1.447 | 0.583-3.591 |
| HBV-DNA, IU/mL, > 2000 | .849 | 0.924 | 0.413-2.071 | .933 | 0.981 | 0.624-1.542 |
| Antiviral therapy, yes | .026 | 0.391 | 0.171-0.896 | .113 | 1.477 | 0.912-2.389 |
| NLR >2.15 | .595 | 1.339 | 0.457-3.927 | .891 | 1.038 | 0.605-1.783 |
| TBIL, µmol/L, >17 | .380 | 1.440 | 0.638-3.248 | .092 | 1.487 | 0.938-2.359 |
| ALB, g/L, ≥35 | .568 | 0.731 | 0.249-2.144 | .298 | 0.727 | 0.399-1.325 |
| ALT, U/L, >80 | .869 | 1.071 | 0.477-2.405 | .996 | 1.001 | 0.635-1.578 |
| PT, seconds, >13 | .837 | 1.102 | 0.436-2.788 | .944 | 1.018 | 0.610-1.700 |
| AFP, µg/L, >400 | .257 | 0.620 | 0.271-1.418 | .290 | 0.783 | 0.498-1.232 |
| PIVKA, mAU/mL, >100 | .940 | 1.058 | 0.245-4.568 | .065 | 0.562 | 0.305-1.036 |
| Surgical therapy, yes | .005 | 0.263 | 0.104-0.663 | .324 | 0.795 | 0.505-1.253 |
| Cirrhosis, yes | .930 | 0.962 | 0.411-2.254 | .479 | 1.184 | 0.742-1.890 |
| Tumour size ≥ 5 cm | .425 | 0.685 | 0.270-1.736 | .232 | 0.731 | 0.438-1.222 |
| Tumour number >3 | <.001 | 4.856 | 2.025-11.646 | .001 | 2.241 | 1.396-3.599 |
| PVTT, Type III | .687 | 1.284 | 0.381-4.332 | .026 | 2.022 | 1.087-3.763 |
| ORR, no | .013 | 3.055 | 1.262-7.396 | .091 | 1.684 | 0.920-3.082 |
| Local treatment, yes | .146 | 0.550 | 0.245-1.231 | .839 | 1.051 | 0.650-1.699 |
| **Abbreviation:** OS, Overall survival; EFS, Event-free survival; HR, Hazard Ratio; CI, Confiden Intenral; ECOG, Eastern Cooperative Oncology Group; HBsAg, hepatitis B surface antigen; HBV-DNA, hepatitis B virus deoxyribonucleic acid; TBIL, total bilirubin; ALB, Albumin; ALT, alanine aminotransferase; PT, prothrombin time; AFP, a-fetoprotein; PIVKA-II, Protein Induced by Vitamin K Ab; NLR, neutrophil to lymphocyte ratio; PVTT, portal vein tumor thrombus; ORR, Objective Response Rate. | | | | | | |
