# Supplementary material for: Prognoses of Patients Treated With Surgical Therapy Versus Continuation of Local-Plus-Systemic Therapy Following Successful Down-Staging of Intermediate-Advanced Hepatocellular Carcinoma: A Multicenter Real-World Study
Source: Oncologist. 2023 Oct 24;29(4):e487–97. doi: 10.1093/oncolo/oyad277 (PMC10994252; doi:10.1093/oncolo/oyad277)
Supplement: oyad277_suppl_Supplementary_Table_S6 [file oyad277_suppl_supplementary_table_s6.docx]

| **Supplement Table 6. Univarate analysis of OS and EFS of BCLC stage B patients who meet the surgical resection criteria after local-plus-systemic therapy** | | | | | | |
| --- | --- | --- | --- | --- | --- | --- |
| **Variable** | **OS** | | | **EFS** | | |
|  | ***P-value*** | **HR** | **95%CI** | ***P-value*** | **HR** | **95%CI** |
| Age, years, >60 | .780 | 0.819 | 0.201-3.336 | .092 | 1.992 | 0.894-4.436 |
| Sex, male | .973 | 1.037 | 0.129-8.351 | .441 | 0.616 | 0.180-2.109 |
| ECOG score, 2/3 | .760 | 0.721 | 0.089-5.870 | .427 | 0.609 | 0.179-2.073 |
| HBsAg, positive | .913 | 0.925 | 0.228-3.748 | .687 | 1.226 | 0.455-3.307 |
| HBV-DNA, IU/mL, > 2000 | .430 | 1.771 | 0.428-7.323 | .317 | 1.535 | 0.663-3.555 |
| Antiviral therapy, yes | .158 | 0.375 | 0.096-1.462 | .206 | 1.644 | 0.761-3.552 |
| NLR >2.15 | .194 | 3.975 | 0.496-31.856 | .067 | 2.368 | 0.942-5.955 |
| TBIL, µmol/L, >17 | .604 | 0.692 | 0.172-2.778 | .577 | 1.255 | 0.566-2.782 |
| ALB, g/L, ≥35 | .450 | 0.434 | 0.050-3.778 | .864 | 0.880 | 0.204-3.799 |
| ALT, U/L, >80 | .619 | 1.382 | 0.387-4.935 | .982 | 1.004 | 0.468-2.153 |
| PT, seconds, >13 | .305 | 2.084 | 0.512-8.474 | .340 | 1.510 | 0.648-3.522 |
| AFP, µg/L, >400 | .347 | 0.537 | 0.147-1.962 | .116 | 0.524 | 0.234-1.172 |
| PIVKA, mAU/mL, >100 | .205 | 0.396 | 0.094-1.660 | .471 | 0.710 | 0.280-1.800 |
| Surgical therapy, yes | .031 | 0.210 | 0.051-0.864 | .633 | 0.818 | 0.360-1.861 |
| Cirrhosis, yes | .948 | 0.955 | 0.237-3.839 | .031 | 2.447 | 1.084-5.526 |
| Tumour size ≥ 5 cm | .311 | 0.481 | 0.116-1.985 | .325 | 0.652 | 0.278-1.529 |
| Tumour number, >3 | .044 | 4.644 | 1.041-20.719 | .047 | 2.447 | 1.011-5.924 |
| Local treatment, yes | .837 | 0.869 | 0.227-3.327 | .081 | 2.329 | 0.901-6.022 |
| **Abbreviation:** OS, Overall survival; EFS, Event-free survival; HR, Hazard Ratio; CI, Confiden Intenral; ECOG, Eastern Cooperative Oncology Group; HBsAg, hepatitis B surface antigen; HBV-DNA, hepatitis B virus deoxyribonucleic acid; TBIL, total bilirubin; ALB, Albumin; ALT, alanine aminotransferase; PT, prothrombin time; AFP, a-fetoprotein; PIVKA-II, Protein Induced by Vitamin K Ab; NLR, neutrophil to lymphocyte ratio; PVTT, portal vein tumor thrombus; ORR, Objective Response Rate. | | | | | | |
