# Supplementary material for: Prognoses of Patients Treated With Surgical Therapy Versus Continuation of Local-Plus-Systemic Therapy Following Successful Down-Staging of Intermediate-Advanced Hepatocellular Carcinoma: A Multicenter Real-World Study
Source: Oncologist. 2023 Oct 24;29(4):e487–97. doi: 10.1093/oncolo/oyad277 (PMC10994252; doi:10.1093/oncolo/oyad277)
Supplement: oyad277_suppl_Supplementary_Table_S7 [file oyad277_suppl_supplementary_table_s7.docx]

| **Supplement Table 7. Multivariable analysis of OS and EFS of BCLC stage B patients who meet the surgical resection criteria after local-plus-systemic therapy** | | | | | | |
| --- | --- | --- | --- | --- | --- | --- |
| **Variable** | **OS** | | | **EFS** | | |
|  | ***P-value*** | **HR** | **95%CI** | ***P-value*** | **HR** | **95%CI** |
| Surgical therapy, yes | .019 | 0.171 | 0.039-0.751 | - | - | - |
| Cirrhosis, yes | - | - | - | .034 | 2.400 | 1.068-5.391 |
| Tumour number, >3 | .029 | 5.872 | 1.204-28.632 | .051 | 2.403 | 0.995-5.802 |
| **Abbreviation:** OS Overall survival; EFS, Event-free survival; HR, Hazard Ratio; CI, Confiden Intenral. | | | | | | |
