# Supplementary material for: Prognoses of Patients Treated With Surgical Therapy Versus Continuation of Local-Plus-Systemic Therapy Following Successful Down-Staging of Intermediate-Advanced Hepatocellular Carcinoma: A Multicenter Real-World Study
Source: Oncologist. 2023 Oct 24;29(4):e487–97. doi: 10.1093/oncolo/oyad277 (PMC10994252; doi:10.1093/oncolo/oyad277)
Supplement: oyad277_suppl_Supplementary_Table_S8 [file oyad277_suppl_supplementary_table_s8.docx]

| **Supplement Table 8. Univarate analysis of OS and EFS of BCLC stage C patients who meet the surgical resection criteria after local-plus-systemic therapy** | | | | | | |
| --- | --- | --- | --- | --- | --- | --- |
| **Variable** | **OS** | | | **EFS** | | |
|  | ***P-value*** | **HR** | **95%CI** | ***P-value*** | **HR** | **95%CI** |
| Age, years, >60 | .416 | 1.600 | 0.515-4.965 | .506 | 0.787 | 0.389-1.593 |
| Sex, male | .791 | 0.818 | 0.184-3.627 | .925 | 1.038 | 0.473-2.280 |
| ECOG score, 2/3 | .454 | 1.790 | 0.390-8.214 | .837 | 1.104 | 0.432-2.817 |
| HBsAg, positive | .870 | 1.184 | 0.155-9.034 | .949 | 0.973 | 0.418-2.266 |
| HBV-DNA, IU/mL, > 2000 | .701 | 0.824 | 0.308-2.210 | .842 | 0.952 | 0.586-1.546 |
| Antiviral therapy, yes | .051 | 0.364 | 0.131-1.006 | .138 | 1.482 | 0.881-2.493 |
| NLR >2.15 | .833 | 1.130 | 0.362-3.522 | .029 | 0.567 | 0.341-0.944 |
| TBIL, µmol/L, >17 | .162 | 2.030 | 0.752-5.480 | .376 | 1.266 | 0.751-2.134 |
| ALB, g/L, ≥35 | .451 | 0.616 | 0.175-2.167 | .401 | 0.757 | 0.395-1.450 |
| ALT, U/L, >80 | .804 | 0.882 | 0.328-2.375 | .882 | 0.964 | 0.593-1.567 |
| PT, seconds, >13 | .719 | 0.792 | 0.223-2.811 | .732 | 1.104 | 0.626-1.949 |
| AFP, µg/L, >400 | .474 | 0.691 | 0.251-1.903 | .998 | 1.001 | 0.617-1.624 |
| PIVKA, mAU/mL, >100 | .093 | 5.699 | 0.750-43.294 | .495 | 0.803 | 0.427-1.509 |
| Surgical therapy, yes | .008 | 0.213 | 0.069-0.662 | .596 | 0.874 | 0.531-1.437 |
| Cirrhosis, yes | .756 | 1.174 | 0.426-3.240 | .906 | 1.031 | 0.623-1.706 |
| Tumour size ≥ 5 cm | .699 | 0.779 | 0.220-2.763 | .812 | 0.929 | 0.504-1.710 |
| Tumour number, >3 | .003 | 4.971 | 1.746-14.153 | .001 | 2.462 | 1.446-4.192 |
| PVTT, Type III | .152 | 2.314 | 0.734-7.294 | .023 | 2.088 | 1.108-3.935 |
| Local treatment, yes | .126 | 0.460 | 0.170-1.243 | .631 | 0.880 | 0.523-1.481 |
| **Abbreviation:** OS, Overall survival; EFS, Event-free survival; HR, Hazard Ratio; CI, Confiden Intenral; ECOG, Eastern Cooperative Oncology Group; HBsAg, hepatitis B surface antigen; HBV-DNA, hepatitis B virus deoxyribonucleic acid; TBIL, total bilirubin; ALB, Albumin; ALT, alanine aminotransferase; PT, prothrombin time; AFP, a-fetoprotein; PIVKA-II, Protein Induced by Vitamin K Ab; NLR, neutrophil to lymphocyte ratio; PVTT, portal vein tumor thrombus; ORR, Objective Response Rate. | | | | | | |
