# Supplementary material for: Prognoses of Patients Treated With Surgical Therapy Versus Continuation of Local-Plus-Systemic Therapy Following Successful Down-Staging of Intermediate-Advanced Hepatocellular Carcinoma: A Multicenter Real-World Study
Source: Oncologist. 2023 Oct 24;29(4):e487–97. doi: 10.1093/oncolo/oyad277 (PMC10994252; doi:10.1093/oncolo/oyad277)
Supplement: oyad277_suppl_Supplementary_Table_S9 [file oyad277_suppl_supplementary_table_s9.docx]

| **Supplement Table 9. Multivariable analysis of OS and EFS of BCLC stage C patients who meet the surgical resection criteria after local-plus-systemic therapy** | | | | | | |
| --- | --- | --- | --- | --- | --- | --- |
| **Variable** | **OS** | | | **EFS** | | |
|  | ***P-value*** | **HR** | **95%CI** | ***P-value*** | **HR** | **95%CI** |
| NLR >2.15 | - | - | - | .003 | 0.434 | 0.251-0.751 |
| Surgical therapy, yes | .026 | 0.269 | 0.085-0.854 | - | - | - |
| Tumour number, >3 | .015 | 3.788 | 1.301-11.030 | <.001 | 2.709 | 1.576-4.657 |
| PVTT, Type III | - | - | - | .002 | 2.965 | 1.504-5.845 |
| **Abbreviation:** OS, Overall survival; EFS, Event-free survival; HR, Hazard Ratio; CI, Confiden Intenral; NLR, neutrophil to lymphocyte ratio; PVTT, portal vein tumor thrombus. | | | | | | |
